# Communication Strategies: The Fuel for Quality Coach-Athlete Relationships and Athlete Satisfaction

**DOI:** 10.3389/fpsyg.2019.02156

**Published:** 2019-09-24

**Authors:** Louise Davis, Sophia Jowett, Susanne Tafvelin

**Affiliations:** ^1^Department of Psychology, Umeå University, Umeå, Sweden; ^2^School of Sport, Exercise and Health Sciences, Loughborough University, Loughborough, United Kingdom

**Keywords:** communication, relationship quality, athlete satisfaction, longitudinal, coach-athlete relationship

## Abstract

The present two-study paper examined the role of communication strategies that athletes use to develop their coach-athlete relationship. Study 1 examined the mediating role of motivation, support, and conflict management strategies between the quality of the coach-athlete relationship and athletes’ perceptions of sport satisfaction. Study 2 examined the longitudinal and mediational associations of communication strategies and relationship quality across two time points, over a 6-week period. Within both studies, data were collected through multi-section questionnaires assessing the studies’ variables. For study 1, structural equation modeling highlighted significant indirect effects for motivation and support strategies between the quality of the coach-athlete relationship and athletes’ experiences of sport satisfaction. For study 2, significant indirect effects were found for the athletes’ perceptions of the quality of the coach-athlete relationship at time 2 between athletes’ use of communication strategies at time point 1 and time point 2. Together these findings provide support for the practical utility of communications strategies in enhancing the quality of the coach-athlete relationship and athlete’s experiences of sport satisfaction. In addition, the findings provide evidence to highlight the potential cyclical relationship between communication and relationship quality across time.

## Introduction

Following major performance success in competitive events (e.g., European, World or Olympic Championships), athletes often and readily acknowledge their coach’s support. This is evident in their post-competition interviews and in the narratives found in autobiographies of high profile athletes (e.g., David Beckham, Paula Radcliffe, Steve Redgrave). Whilst athletes form many significant relationships over the course of their sporting career, the relationship they form with their coach is key to their sporting development and performance success (Jowett and Shanmugam, 2016). Athletes’ relationship with their coaches are often characterized as task-focused that aim to provide a purposeful and meaningful social situation from which coaches and athletes support one another to achieve goals that are relevant to them and their relationship (Jowett, 2017). Over time, this unique partnership is thought to be centered around both the coach and the athlete (“coach-athlete centered”) promoting inclusivity that is mutually empowering (Jowett, 2017). Such a coach-athlete centered approach, whereby coaches and athletes are meaningfully connected is more likely to function as a medium that motivates, assures, satisfies, comforts, and supports toward enhancing their sport experience and performance, as well as overall well-being.

Over the past two decades, the quality and functions of the coach-athlete relationship have been studied (see, e.g., Jowett and Meek, 2000; Wylleman, 2000; Poczwardowski et al., 2002; LaVoi, 2004). Jowett (2007) defined the coach-athlete relationship as a social situation within which a coach’s and an athlete’s feelings of *closeness* (i.e., an emotional connection reflected in trust, like, respect), thoughts of *commitment* (i.e., motivation to maintain a close relationship over time), and behaviors of *complementarity* (i.e., behaviors reflected in interactions that are responsive, relaxed, and friendly) are mutually and causally interconnected. Closeness, commitment and complementarity form the 3Cs of the 3 + 1Cs model of the coach-athlete relationship quality (Jowett, 2007, 2017). Co-orientation reflects the +1 element of this model and refers to the degree to which athlete’s and the coach’s perceptions are interconnected. Co-orientation contains two perspectives: the direct perspective and the meta perspective; both of which are conceptually diverse concepts (Kenny, 1994; Jowett, 2007). For example, the direct perspective reflects how the athlete/coach feels, thinks and behaves toward the other (e.g., ‘I trust my coach/athlete’) whilst the meta perspective is reflected in the way in which the athlete or the coach perceives how the other thinks, feels and behaves (e.g., ‘My coach/athlete trusts me’).

Evidence has shown that the quality of the coach-athlete relationship associates with important performance-related and well-being outcomes including sport and relationship satisfaction (Lorimer and Jowett, 2009; Davis and Jowett, 2014), motivation (Adie and Jowett, 2010; Felton and Jowett, 2013), team cohesion (Jowett and Chaundy, 2004), collective efficacy (Hampson and Jowett, 2014), well-being indicators (Felton and Jowett, 2013), and physical and cognitive performance (Davis et al., 2018). Recently, research has also highlighted negative outcomes associated with poor quality coach-athlete relationships including interpersonal conflict (Wachsmuth et al., 2018) and athlete burnout (Isoard-Gautheur et al., 2016; Davis et al., 2018).

While the quality of the coach-athlete relationship is linked with both positive and negative outcomes, it is important to understand the mechanisms by which the relationship quality associates with such important outcomes. Jowett and Poczwardowski (2007) through their integrated research model acknowledged the importance of interpersonal communication as a key factor that affects and is affected by the quality of the coach-athlete relationship. Furthermore, research revolving around talent development has highlighted that open and honest communication is a vital feature as it allows coaches and athletes to share information (e.g., goals, individualized programs) and build trust (e.g., Martindale et al., 2007). More recently, Gilbert (2017) explained “the most effective coaching strategy for building and sustaining quality coach-athlete relationship is communication” (p. 78). Therefore, in this study, communication is thought to be a potential mechanism that may be capable to transfer the effects of coach-athlete relationship quality onto both interpersonal (e.g., relationship satisfaction) and intrapersonal outcomes (e.g., sport satisfaction, motivation, sport performance) (see Jowett, 2007; Lorimer and Jowett, 2014). Although, there is still no empirical evidence to truly substantiate this claim, Rhind and Jowett (2010, 2011, 2012) captured the communication strategies coaches and athletes use to keep the relationship in a specified state (cf. Dindia and Canary, 1993) and to enhance the relationship quality (cf. Canary and Stafford, 1992). Guided by Dindia and Canary’s (1993) description of relationship maintenance as well as Stafford and Canary (1991) measure of relationship maintenance, Rhind and Jowett (2011) proposed the COMPASS model and associated measures (i.e., CARM-Q) in order to identify and measure relationship maintenance strategies (i.e., communication strategies) within the context of the coach-athlete relationship. Accordingly, the COMPASS model (Rhind and Jowett, 2012) contains seven key communication strategies: *Conflict management* reflects coaches’ and athletes’ efforts to identify, discuss, resolve, and monitor potential areas of disagreement; *openness* includes efforts to engage in opens lines of communication; *motivation* highlights both coaches’ and athletes’ efforts to develop a partnership that is both rewarding, active, and ambitious in providing reasons for each member to stay in the relationship; *preventative* underlines efforts to discuss expectations, rules, and roles and what should happen if these are not met; *Support* is reflected in coaches’ and athletes’ helping one another through difficult and or challenging times; and *Social networks* reflect communication strategies that create opportunities to develop strong bonds with significant others (e.g., parents, friends, training squad, managers, sports science agents) that can prevent the coach and athlete operating in a “bubble” detached from reality.

Limited research has investigated the associations between communication strategies (via the COMPASS model) and coach -athlete relationship quality (via the 3CS). For example, Rhind and Jowett (2011, 2012) developed and administered the Coach-Athlete Relationship Maintenance Questionnaire (CARM-Q) to a sample of athletes across two studies and found that those athletes, who reported higher levels of closeness, also reported greater use of communication strategies and more specifically, higher levels of open channels of communication and more engagement with their wider social network. Athletes who reported greater levels of commitment with their coach, also reported greater use of motivational strategies (i.e., providing reasons to stay in the current relationship), assurance (i.e., showing their relationship member they can count on them) and support strategies (i.e., providing support to each other during times of need). Finally, those athletes who reported greater levels of complementarity shown to engage more with the use of conflict management strategies (i.e., managing areas of disagreement and conflict) and preventative strategies (i.e., discussing expectations) within the context of the coach-athlete relationship. Rhind and Jowett (2012) suggested that the absence of such strategies may be aligned with lack of connection as expressed through emotional distance, unwillingness to continue the relationship and difficulty in working together, as well as hostility, dissimilarity and discord (see Wachsmuth et al., 2018).

Overall, initial empirical findings highlight the utility of the COMPASS model as an important psychological process of relationship development and maintenance. Thus, more research is required to overcome both methodological and conceptual limitations that have been presented to date. For example, there is scope to examine communication as a mechanism that transfers the effects of relationship quality onto sporting outcomes (Jowett and Shanmugam, 2016). Further, longitudinal research to assess temporal patterns and associations between communication strategies and perceptions of relationship quality would be of benefit. Lastly, research is also warranted to test the bi-directional nature of interpersonal communication as outlined in Jowett and Poczwardowski (2007) model. This research has the potential to contribute to the conceptual assumptions that have been made over the years (see, e.g., Jowett, 2007) by generating knowledge and understanding of the interplay between coach-athlete communication and relationship quality. In turn, this generated knowledge and understanding would have the potential to inform interventions research studies as well as coach educational programs that aim to improve the efficacy of coach-athlete interactions and the effectiveness of coaching more generally.

### The Present Study

The quality of relationships coaches and athletes develop and maintain with one another creates a social situation that can be viewed as positive (rewarding, supportive, motivating) or negative (disappointing, unhelpful, uninspiring). Research findings suggest that coaches and athletes who find themselves in social situations that are positive are more likely to achieve their performance-related outcomes (Jowett and Chaundy, 2004; Hampson and Jowett, 2014) but also outcomes associated with their well-being (Felton and Jowett, 2015; Davis et al., 2018) Correspondingly, athletes and coaches who find themselves in positive quality relationships are also more likely to have engaged in interpersonal communication that is constructive and helpful (Rhind and Jowett, 2012). That said, communication strategies have not been examined as a mechanism by which relationship quality associates with important outcomes. Communication (via COMPASS) has the capacity to empower, energize and fuel the effects of the coach-athlete relationship (via 3Cs) including the broader social environment within which the relationship is embedded, on important outcomes such as satisfaction with sport (Rhind and Jowett, 2012; Wachsmuth et al., 2018). Therefore, study 1 of this two-study paper examined the mechanisms by which communication strategies transfer the effects of the coach-athlete relationship quality onto dimensions of sport satisfaction employing a mediational research design. For the purpose of this study, three communication strategies were utilized from the COMPASS model (Rhind and Jowett, 2010) which included: motivational, support, and conflict management strategies. Rhind and Jowett (2012) found that these three strategies were associated with at least one dimension of quality coach-athlete relationships (i.e., 3Cs: Closeness, Commitment, and Complementarity). It was hypothesized that the communication strategies of motivation, support and conflict management would transfer the effects of relationship quality as defined by the 3Cs (both direct and meta perceptions) to athletes’ perceptions of satisfaction with personal treatment, performance, as well as training and instruction.

Taking into account, that communication is viewed a key factor that affects and is affected by the quality of the coach-athlete relationship (Jowett and Poczwardowski, 2007), the aims of study 2 was to extend Study 1 by examining the causal associations between the communication strategies of motivation, support and conflict management and relationship quality. A longitudinal research design was employed that contained data collection across two time points. It was hypothesized that athletes’ perceptions of the quality of the coach-athlete relationship at time point two would mediate their perceptions of communication at time point one and time point two. Subsequently, this study not only explores communication as a determinant variable of relationship quality but also explores whether relationship quality is a likely determinant variable of interpersonal communication. This potential reciprocity in the association between these two variables would extend research (Martindale et al., 2007; Gilbert, 2017) and confirm conceptual assumptions (Jowett and Poczwardowski, 2007).

## Study 1

### Methods

#### Participants

A sample of 182 female and male athletes (*M*_*age*_ = 21.1 years, *SD* = ± 4.1) participated in this study. Of the 182 athletes, 38.5% were female and 61.5% were male. Athletes represented a variety of individual sports (e.g., swimming, gymnastics, tennis, rowing, dance, and athletics) and team sports (e.g., football, hockey, water polo, volleyball, squash, lacrosse, and rugby) and competed at International (8.2%) National (13.2%), Regional (12.1%), and Club (66.5%) levels. Club level athletes were those who competed for their local sports clubs in comparison to those athletes who competed at regional, national, and international championships. Athletes had participated in their sport for an average of 8.2 years (*SD* = ± 4.1).

#### Measurements

##### Coach-athlete relationship quality

The Coach-Athlete Relationship Questionnaire both direct and meta-perspective versions (CART-Q; Jowett and Ntoumanis, 2004; Jowett, 2009a) was employed. The 11-item direct perspective of the CART Q has 4 items that represented closeness (e.g., I trust my coach/athlete), 3 items that represented comittment (e.g., I am committed to my coach/athlete), and 4 items that represented complementarity (e.g., I am responsive to his/her efforts). The 11-item meta-perspective of the CART-Q contains the same items though re-worded to reflect athletes’ meta-perceptions closeness (e.g., My coach/athlete likes me), commitment (e.g., My coach/athlete is committed to me), and complementarity (e.g., My coach/athlete is responsive to my efforts during training). The response scale ranged from 1 (“Strongly Disagree”) to 7 (“Strongly Agree”). Previous studies have displayed sound psychometric properties of the CART-Q (see Jowett and Ntoumanis, 2004; Jowett, 2009a, b). The quality of the coach-athlete relationship from an athlete direct and meta-perspective was represented by a global score each. This approach has been utilized in previous studies (e.g., Jowett, 2008; Lorimer and Jowett, 2009; Davis and Jowett, 2013).

##### Coach-athlete relationship maintenance strategies

The Coach-Athlete Relationship Maintenance Questionnaire (CARM-Q; Rhind and Jowett, 2012) was employed to assess the three subscales: 5 items assessed conflict management (e.g., I am understanding during disagreements); 5 items assessed motivational strategies (e.g., I show my coach I am motivated to work hard with my coach); and 3 items assessed support (e.g., I give my coach support when things are not going well). Respondents indicated their agreement with the items on a seven-point scale from 1 ‘strongly disagree’ to 7 ‘strongly agree.’ CARM-Q’s psychometrics properties have been found to be sound in previous studies (e.g., Rhind and Jowett, 2012).

##### Athlete satisfaction

The Athlete Satisfaction Questionnaire (ASQ; Riemer and Chelladurai, 1998) was employed to assess three facets of athlete satisfaction: 3 items assessed satisfaction with individual performance (e.g., “I am satisfied with the improvement in my skill level thus far”); 3 items assessed satisfaction with training and instruction (e.g., “I am satisfied with the training and instruction I have received from the coach this season”); and 5 items assessed satisfaction with personal treatment (e.g., “I am satisfied with the level of appreciation my coach shows when I do well”). Participants rated the extent to which they felt satisfied with each item on a seven-point Likert-type scale ranging from 1 (not at all satisfied) to 7 (extremely satisfied). The ASQ items have sound psychometric properties (see Riemer and Chelladurai, 1998; Davis and Jowett, 2010).

#### Procedure

Ethical approval for the present study was granted by the ethics committee of the first author’s previous institution, Northumbria University. Athletes were then recruited via existing contacts and sports clubs across the United Kingdom. Contact was made via email, where the purpose, procedure and voluntary nature of the study was explained. A convenient time to visit the participant prior to or at the end of a training session was discussed to elicit their written consent and for them to participate in the study. On gaining written consent, athletes were administered a multi-section questionnaire that contained demographic information (e.g., age, gender, sport level, and relationship length) and the questionnaires (CART-Q, CARM-Q, ASQ). APA guidelines regarding anonymity and confidentiality were followed and guaranteed. Upon completion, athletes were verbally debriefed and thanked for their participation. The entire procedure lasted approximately 15 min.

#### Data Analysis

Firstly, descriptive statistics and bivariate correlations were performed. Structural equation modeling (SEM) with maximum likelihood (ML) estimation was then employed to examine the study hypotheses. We used the recommended procedure for testing mediational models in SEM and estimated both the direct and indirect effects of relationship quality on the three dimensions of athlete satisfaction by employing bootstrapping procedures to assess 95% bias-corrected (BC) confidence intervals (CIs) of these effects (Rucker et al., 2011). Goodness of fit indices including the chi-square statistic, the comparative fit index (CFI), the standardized root mean-square residual (SRMR), and the root mean square error of approximation (RMSEA) were used to evaluate the estimated models. Conventional cut-off criteria were used to estimate adequate fit (CFI > 0.90, SRMR and RMSEA < 0.08; Kline, 2010) and very good fit (CFI > 0.95; SRMR < 0.08 and RMSEA < 0.06; Hu and Bentler, 1999). Mediation was assessed by examining indirect effects using a 95% BC CI. This allowed us to interpret how accurate the sample statistic reflected the population parameters (Preacher and Kelley, 2011).

### Results

#### Descriptive Statistics

Table 1 presents the means, standard deviations, alpha reliability coefficients and bivariate correlations for all variables investigated in this study. On average, the sample reported higher levels of perceived relationship quality from both a direct and meta perspective as well as moderate to higher levels of sport satisfaction. For the most part, the sample also reported the use of maintenance strategies within their coach-athlete relationship as moderate to high. Bivariate correlations were computed to assess the degree and direction of the relationship between the direct and meta perspectives of the quality of the coach-athlete relationship, the 3 relationship maintenance strategies and the 3 sport satisfaction dimensions. Statistically significant correlations were found between relationship quality (direct and meta), relationship maintenance strategies and sport satisfaction dimensions. Furthermore, the directions of the correlations were as expected.

**TABLE 1:** Descriptive statistics, Cronbach’s alpha coefficients and inter correlations for all main variables in the study.

**Variables**	***M***	***SD***	**α**	**1**	**2**	**3**	**4**	**5**	**6**	**7**	**8**
**Athlete variables**											
(1) Direct relationship quality	5.50	1.1	0.95	−							
(2) Meta relationship quality	5.49	1.0	0.94	0.88^∗∗^	−						
(3) Conflict management	5.37	1.1	0.88	0.38^∗∗^	0.46^∗∗^	−					
(4) Motivational	5.93	0.9	0.91	0.55^∗∗^	0.58^∗∗^	0.34^∗∗^	−				
(5) Support	4.25	1.8	0.90	0.51^∗∗^	0.53^∗∗^	0.34^∗∗^	0.25^∗∗^	−			
(6) Performance satisfaction	5.34	1.2	0.86	0.60^∗∗^	0.60^∗∗^	0.33^∗∗^	0.53^∗∗^	0.48^∗∗^	−		
(7) Training satisfaction	5.23	1.2	0.88	0.59^∗∗^	0.54^∗∗^	0.36^∗∗^	0.46^∗∗^	0.46^∗∗^	0.48^∗∗^	−	
(8) Treatment satisfaction	5.39	1.3	0.87	0.55^∗∗^	0.57^∗∗^	0.34^∗∗^	0.39^∗∗^	0.55^∗∗^	0.74^∗∗^	0.76^∗∗^	−

*^∗∗^*p* significant at 0.01.*

#### Mediation Analyses

Following the procedures outlined by Anderson and Gerbing (1988), two measurement models (one with direct relationship quality and another with meta relationship quality) were examined prior to estimating the structural models. The measurement models consisted of seven intercorrelated latent variables: direct or meta relationship quality, the three relationship maintenance strategies (conflict management, support, and motivational) and the three dimensions of sport satisfaction (satisfaction with individual performance, training and instruction, and personal treatment). Both models demonstrated an adequate fit to the data, model fit for the model with direct relationship quality was χ^2^(231) = 398.11, *p* < 0.001, CFI = 0.95, RMSEA = 0.06, 95% CI (0.052, 0.073), SRMR = 0.06, and model fit for the model with meta relationship quality was χ^2^(231) = 380.24, *p* < 0.001, CFI = 0.96, RMSEA = 0.06, 95% CI (0.049, 0.073), SRMR = 0.06.

**FIGURE 1:**
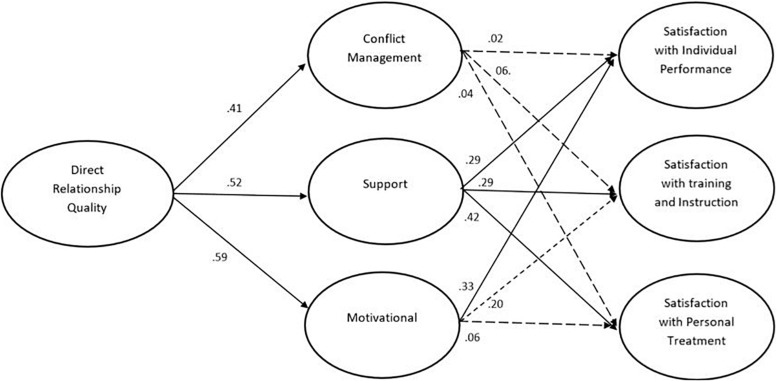
The mediation model describing mediation of relational maintenance strategies in the link between the direct perspective of coach-athlete relationship quality and athlete satisfaction, Only standardized co-efficients are presented, The estimates between relationship quality and sport satisfaction was omitted for clarity.

Next, the two structural models were estimated. The first model included direct paths from direct relationship quality on athlete satisfaction (in terms of individual performance, training and instruction, and personal treatment) as well as indirect relationships mediated by conflict management, support, and motivational strategies respectively (see Figure 1). The model provided an adequate fit to the data: χ^2^(231) = 398.11, *p* < 0.001, CFI = 0.95, RMSEA = 0.06, 95% CI (0.052, 0.073), SRMR = 0.06. As presented in Figure 1, direct relationship quality was significantly related to all three mediators including conflict management (β = 0.41, *p* > 0.00), support (β = 0.52, *p* > 0.00), and motivational (β = 0.59, *p* > 0.00), as well as with the outcomes in terms of individual performance (β = 0.31, *p* > 0.001), satisfaction with training and instruction (β = 0.34, *p* > 0.00), and satisfaction with personal treatment (β = 0.32, *p* > 0.00). Among the mediators, conflict management was unrelated with the athlete satisfaction scales, whilst the motivational strategy was significantly related to individual performance (β = 0.33, *p* > 0.00) and satisfaction with training and instruction (β = 0.20, *p* > 0.01). Support was significantly related to all three outcomes including individual performance (β = 0.29, *p* > 0.00), satisfaction with training and instruction (β = 0.29, *p* > 0.00), and satisfaction with personal treatment (β = 0.42, *p* > 0.00).

The indirect effects of direct relationship quality on the three athlete satisfaction scales through the three maintenance strategies were all significant, including on individual performance (β = 0.40; 95% BC CI [0.20, 0.59], *p* = 0.000) satisfaction with training and instruction (β = 0.32; 95% BC CI [0.10, 0.52], *p* = 0.000), and satisfaction with personal treatment (β = 0.31; 95% BC CI [0.06, 0.52], *p* = 0.005). As a way to compare the three mediators we also examined specific indirect effects of direct relationship quality through conflict management, support, and motivational strategies. The analyses showed that support mediated specific indirect effects on all three outcomes including individual performance (β = 0.15; 95% BC CI [0.08, 0.28], *p* = 0.000) satisfaction with training and instruction (β = 0.15; 95% BC CI [0.04, 0.28], *p* = 0.001), and satisfaction with personal treatment (β = 0.22; 95% BC CI [0.13, 0.40], *p* = 0.000). Motivational strategies mediated a specific indirect effect on satisfaction with individual performance (β = 0.19; 95% BC CI [0.08, 0.41], *p* = 0.002), while all specific indirect effects through conflict management were insignificant.

**FIGURE 2:**
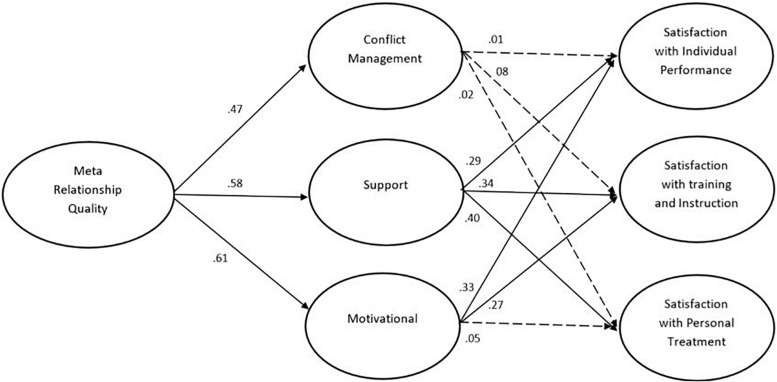
The mediation model describing mediation of relational maintenance strategies in the link between the meta perspective of coach-athlete relationship quality and athlete satisfaction. Only standardized co-efficients are presented. The estimates between relationship quality and sport satisfaction was omitted for clarity.

The second structural model was similar to the first model but included the meta relationship quality perspective instead of direct relationship quality (see Figure 2). The model provided an adequate fit to the data: χ^2^(231) = 380.24, *p* < 0.001, CFI = 0.96, RMSEA = 0.06, 95% CI (0.049, 0.070), SRMR = 0.06. Meta relationship quality was significantly related to the mediators: conflict management (β = 0.47, *p* < 0.00), support (β = 0.58, *p* < 0.00), and motivational (β = 0.61, *p* < 0.00). In contrast to the first model, meta relationship quality was unrelated to satisfaction with training and instruction, but significantly related to satisfaction with individual performance (β = 0.27, *p* < 0.01) and satisfaction with personal treatment (β = 0.34, *p* < 0.001). Again, conflict management was unrelated to all subscales of athlete satisfaction, and motivational was related to two of subscales including satisfaction with individual performance (β = 0.35, *p* < 0.00) and satisfaction with training and instruction (β = 0.27, *p* < 0.001). Also similar to the first model, support was significantly related to all three athlete satisfaction scales including satisfaction with individual performance (β = 0.29, *p* < 0.00), satisfaction with training and instruction (β = 0.34, *p* < 0.00), and satisfaction with personal treatment (β = 0.40, *p* < 0.00).

In line with the first model, the indirect effects of meta relationship quality on the three athlete satisfaction scales through the three maintenance strategies were significant, including on satisfaction with individual performance (β = 0.42; 95% BC CI [0.20, 0.69]) satisfaction with training and instruction (β = 0.42; 95% BC CI [0.19, 0.70]), and satisfaction with personal treatment (β = 0.31; 95% BC CI [0.08, 0.53]). We then examined specific indirect effects through conflict management, support, and motivational strategies respectively. In line with our findings of direct relationship quality, the analyses showed that support mediated specific indirect effects of meta relationship quality on all three outcomes including individual performance (β = 0.19; 95% BC CI [0.08, 0.31]) satisfaction with training and instruction (β = 0.26; 95% BC CI [0.09, 0.36]), and satisfaction with personal treatment (β = 0.26; 95% BC CI [0.12, 0.41]). Further, the motivational strategies mediated two specific indirect effects including on individual performance (β = 0.23; 95% BC CI [0.09, 0.43]) and satisfaction with training and instruction (β = 0.17; 95% BC CI [0.04, 0.36]). Again, all specific indirect effects through conflict management were insignificant.

### Discussion

This study examined the role of communication strategies as mediators between athletes’ perceptions of the quality of their coach-athlete relationship and important outcomes (i.e., perceptions of athlete sport satisfaction). It was hypothesized that motivation, support, and conflict management strategies would transfer the effects of coach-athlete relationship quality onto athletes’ feelings of satisfaction with their training and instruction, personal treatment, and individual performance. The findings highlight significant direct and indirect effects. Relationship quality positively predicted athlete satisfaction (in terms of individual performance, training and instruction, as well as personal treatment) and all three forms of communication strategies (support, motivational, and conflict management). These findings are consistent with previous research (Martindale et al., 2007; Davis and Jowett, 2010; Rhind and Jowett, 2011, 2012; Davis et al., 2018). Findings would seem to suggest that athletes with quality relationships characterized by high levels of trust, respect, appreciation and commitment (or a strong intention to maintain a close long-term relationship), as well as co-operation where there is reassurance and support as well as approachability and responsiveness are more likely to experience high levels of satisfaction as this pertains to how they are treated, trained, and instructed by coaches as well as how they perform. Furthermore, the findings suggest that communication strategies are a mechanism by which relationship quality and satisfaction are associated. Subsequently, relationship quality and satisfaction are associated because athletes in good quality relationships are more likely to be able to manage conflict, to express their motivation and more readily show their support.

Findings from the bootstrap analysis revealed interesting results for the indirect effects indicating variation of meditation effects. For example, the utility of support strategies was able to fully mediate the relationship between coach-athlete relationship quality and athletes’ satisfaction. Support communicates one’s desire to help, value and care for another and hence may be a central psychological and interpersonal process (see Rees et al., 2016). Secondly, motivational strategies mediated the association between coach-athlete relationship quality and satisfaction with performance, training, and instruction. However, the indirect effects of motivational strategies on the association between coach-athlete relationship quality and satisfaction with personal treatment were non-significant. Put together these findings may underline the task-focused nature of the coach-athlete relationship whereby athletes who connect with their coaches are motivated and hence more successful and competent in their sport (Olympiou et al., 2008; Jowett and Shanmugam, 2016). The motivational properties of the coach-athlete relationship have been found in previous research (e.g., Adie and Jowett, 2010; Jowett, 2017).

The indirect effects of conflict management strategies on the association between coach-athlete relationship quality and all three dimensions of sport satisfaction were non-significant. Therefore, it appears that when athletes have good quality relationships with their coach the mechanism of managing conflict may not be activated. It would be useful to investigate whether conflict management is an active process when relationship quality is poor. Moreover, it is possible that athletes perceive the use of conflict management strategies as an element or process related to the role and responsibilities of the coach (Wachsmuth et al., 2017). Nonetheless, conflict management may be an important process for athletes to understand and engage in especially during conflict episodes and when relationship quality suffers (which may be a conflict in itself).

These findings extend previous research (e.g., Rhind and Jowett, 2012) by demonstrating how the use of relationship maintenance strategies can transfer the effects of relationship quality onto athletes’ experience of satisfaction. The findings generate practical and theoretical information. Practical information in that it helps us identify strategies to use in an intervention to benefit athletes’ interactions with their coaches and their levels of satisfaction. Theoretical information in that it helps us substantiate theoretical or conceptual assumptions put forward in the literature. Future research directions could examine the efficacy of intervention programs whereby communication strategies are systematically and deliberately implemented to enhance both relationship quality and its outcomes. Knowledge regarding which strategies are associated with relationship quality and important outcomes would be advantageous for both theory and practice; the present study provides a strong basis to continue this line of research.

In consideration of the dyadic nature of the coach-athlete relationship and the move toward assessment of the dyad as a unit of analysis (Lorimer and Jowett, 2009; Davis et al., 2013), it would be practically useful to carry out a study involving both members of the relationship. Additionally, researchers may wish to explore potential differences in the utility of communication strategies across a variety of moderating variables including individual characteristics (e.g., age, gender), relationship (e.g., partnership length, typical versus atypical dyads), and environmental factors (e.g., different sports, cultures, competitive levels). Specifically, this study predominately recruited club level athletes, therefore limiting the findings to other competitive levels. Collectively, future research may offer both theoretical and practical knowledge for coaches, athletes, and applied sport psychology practitioners.

In summary, the findings of this study suggest that support and motivational strategies have a positive association with the quality of the coach athlete relationship and athlete satisfaction directly or indirectly. That said, past research has suggested that perceptions of relationship quality and the use of maintenance strategies may change over time and be bi-directional in nature (Jowett and Poczwardowski, 2007; Rhind and Jowett, 2011). Specifically, whilst study 1 has facilitated an understanding that the communication perceived by the athletes was affected by the quality of their coach-athlete relationship; it is also possible that the use of communication strategies may also present as building blocks toward developing and maintaining coach-athlete relationships over time. In light of this, carrying out research that assesses communication strategies alongside perceptions of relationship quality over time would provide a better understanding of temporal patters that could potentially inform intervention programs while understanding the reciprocal nature of these relationship variables. Therefore, the purpose of study 2 was to examine the longitudinal and mediational associations of communication strategies and relationship quality across two time points.

## Study 2

### Methods

#### Participants

A comprised sample of 107 female and male athletes (*M*_*age*_ = 21 years, *SD* = ±2.9) were recruited for participation in this study. Of the 107 athletes, 62.6% of athletes were female and 37.4% were male. Athletes represented a variety of individual sports (e.g., swimming, Archery tennis, athletics) and team sports (e.g., football, netball, basketball, and rugby) and competed at various levels including International (1.9%) National (3.7%), Regional (11.2%), and Club (83.2%) levels. Club level athletes were those who competed for their local sports clubs in comparison to those athletes who competed at regional, national and international championships. Athletes had a mean relationship length of 3.8 years (*SD* = ±1.5).

#### Measurements

##### Coach-athlete relationship quality

The Coach-Athlete Relationship Questionnaire both direct and meta-perspective versions (CART-Q; Jowett and Ntoumanis, 2004; Jowett, 2009a) was employed. The 11-item direct perspective and the corresponding 11-item meta-perspective version of the CART-Q. The response scale ranged from 1 (“Strongly Disagree”) to 7 (“Strongly Agree”). The quality of the coach-athlete relationship from an athlete direct and meta-perspective was represented by a global score each. This approach was utilized in Study 1 and in previous studies (e.g., Jowett, 2008; Lorimer and Jowett, 2009; Davis and Jowett, 2010).

##### Coach-athlete relationship maintenance strategies

The Coach-Athlete Relationship Maintenance Questionnaire (CARM-Q; Rhind and Jowett, 2012) was employed to assess three subscales: 5 items assessed conflict management (e.g., I am understanding during disagreements); 5 items assessed motivational strategies (e.g., I show my coach I am motivated to work hard with my coach); and 3 items assessed support (e.g., I give my coach support when things are not going well). Respondents indicated their agreement with the items on a seven-point scale from 1 ‘strongly disagree’ to 7 ‘strongly agree.’ CARM-Q’s psychometrics properties have been found to be sound in previous studies (e.g., Rhind and Jowett, 2012).

#### Procedure

Ethical approval for this study was granted by the ethics committee of the second author’s institution. Athletes were then recruited via existing contacts and sports clubs across the United Kingdom. The purpose, procedure and voluntary nature of the study were explained and APA guidelines regarding anonymity and confidentiality were followed. Informed consent was obtained from all athletes prior to commencing data collection. Athletes were administered a multi-section questionnaire twice separated by 6 weeks. A 6-week interval was considered sufficient for change to occur. This period was decided based on pragmatic and theoretical considerations. For example, a coach we worked with did not allow data collection passed a certain date. For all participants, this was a busy training and competitive time where interactions between coaches and athletes were rich. Researchers from different disciplines of psychology (health and occupational psychology) have touched upon issues surrounding the length of time between time points. Toon and Kompier (2014) explained that the length of time between time points should correspond with the underlying “‘true” causal lag’ so that the antecedent has sufficient time to affect the outcome variable but at the same time is not too long for the effects to disappear. Moreover, Watson (1998) highlighted that the longer the time between points, the greater the difficulty in retaining the subjects. The questionnaire for both time points contained demographic information (e.g., age, gender, sport level, and relationship length) as well as the CART Q and the three subscales from the CARM-Q. Athletes were also asked to provide a unique code so that their questionnaires were matched at the end of the data collection period. Athletes were asked to complete the questionnaires prior to a training session, in the presence of a research assistant. This process took approximately 15 min on each occasion. Upon completion, athletes were debriefed and thanked for their participation.

#### Data Analysis

Firstly, descriptive statistics and bivariate correlations were performed. To examine whether relationship quality at time 2 predicted longitudinal changes in communication, SEM (using Mplus 7.31) with ML estimation was employed. Before testing the structural models, longitudinal measurement models were specified. In line with current recommendations (Widaman et al., 2010; Little, 2013), configural, metric (factor loadings), and scalar (factor loadings and intercepts) invariance was tested across time in the measurement models. We used change in the comparative fit index (ΔCFI) as the goodness of fit index when comparing the models. A decrease equal to or greater than −0.01 in CFI is considered as an indication of non-invariance (Cheung and Rensvold, 2002). If metric, but not scalar, invariance is reached, partial measurement invariance can be established (Steenkamp and Baumgartner, 1998) which is needed to make valid inferences about the differences between latent factor means in the model (Byrne et al., 1989). Goodness of fit indices including the chi-square statistic, CFI, SRMR, and RMSEA were used to evaluate the estimated models. Conventional cut-off criteria were used to estimate benchmarks for adequate fit (CFI > 0.90, SRMR and RMSEA < 0.08; Kline, 2010) and excellent fit (CFI > 0.95; SRMR < 0.08 and RMSEA < 0.06; Hu and Bentler, 1999). Mediation was assessed by examining indirect effects using a 95% BC CIs, which allowed us to interpret how accurate the sample statistic reflected the population parameters (Preacher and Kelley, 2011).

### Results

#### Descriptive Statistics

Table 2 presents the means, standard deviations, alpha reliability coefficients, and bivariate correlations for all three main variables investigated in this study. On average, the sample reported moderate to high levels of perceived direct and meta relationship quality at time 2 as well as moderate levels of communication at time 1 and time 2. As expected, statistically significant correlations were found between relationship quality (meta and direct perspectives) at time 1 and communication at time 1 and communication at time 2.

**TABLE 2:** Descriptive statistics, Cronbach’s alpha coefficients and inter correlations for all main variables in the study.

**Variables**	***M***	***SD***	**α**	**1**	**2**	**3**	**4**
**Athlete variables**							
(1) Direct relationship quality2	5.34	1.14	0.95	−			
(20 Meta relationship quality2	5.43	1.15	0.97	0.91^∗∗^	−		
(3) Communication strategies 1	4.47	0.96	0.91	0.79^∗∗^	0.76^∗∗^	−	
(4) Communication strategies 2	4.70	0.93	0.91	0.81^∗∗^	0.80^∗∗^	0.96^∗∗^	−

*^∗∗^*p* significant at 0.01.*

#### Mediation Analysis

First, we established if we had measurement invariance over time in the communication measure by examining increasingly restricted measurement models including our two communication measures over time as well as relationship quality (direct or meta). The analyses indicated that we had metric, or partial, measurement invariance with invariant loadings but not intercepts which is sufficient for examining relationships between latent factors over time (Byrne et al., 1989). Model fit for the measurement model including direct relationship quality was adequate: χ^2^(21) = 49.09, *p* < 0.001, CFI = 0.98, RMSEA = 0.11, 95% CI (0.071, 0.153), SRMR = 0.03, as well as for the model with meta relationship quality; χ^2^(21) = 49.33, *p* < 0.001, CFI = 0.98, RMSEA = 0.11, 95% CI (0.072, 0.153), SRMR = 0.03.

**FIGURE 3:**
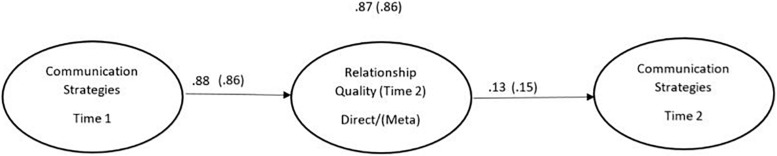
The longitudinal model describing relationship quality at time 2 as a mediator between communication strategies at time 1 and communication strategies at time 2. Only standardized co-efficients are presented and both models (i.e., direct and meta) are presented in this figure for simplicity. Meta Perspective are presented in bracktes.

In the next step, we tested our two structural models, with either direct or meta relationship quality as a mediator between Communication at Time 1 and Communication at Time 2 (see Figure 3). The model with direct relationship quality as a mediator had adequate fit: χ^2^(21) = 49.09, *p* < 0.001, CFI = 0.98, RMSEA = 0.11, 95% CI (0.071, 0.153), SRMR = 0.03. Communication at Time 1 was positively related to direct relationship quality at Time 2 (β = 0.88, *p* < 0.001), as well as with Communication at Time 2 (β = 0.87, *p* < 0.001). Further, direct relationship quality at Time 2 was positively related to Communication at Time 2 (β = 0.13, *p* < 0.04). The indirect effect of Communication at Time 1 on Communication at Time 2 through direct relationship quality was significant (β = 0.11; 95% BC CI [0.001, 0.27]).

The second model, with meta relationship quality at time 2 as a mediator, also had adequate fit: χ^2^(21) = 49.33, *p* < 0.001, CFI = 0.98, RMSEA = 0.11, 95% CI (0.072, 0.153), SRMR = 0.03. Communication at Time 1 was positively related to meta relationship quality at time 2 (β = 0.86, *p* < 0.001), as well as Communication at Time 2 (β = 0.86, *p* < 0.001). Also, meta relationship quality was positively related to Communication at Time 2 (β = 0.15, *p* < 0.01). Further, the indirect effect of Communication at Time 1 on Communication at Time 2 through meta relationship quality was significant (β = 0.13; 95% BC CI [0.04, 0.28]).

### Discussion

The aim of study 2 was to examine the associations between communication strategies and coach-athlete relationship quality over time. Using a full cross-lagged design with two measurement points spanning a 6-week period, we hypothesized that athletes’ perceptions of the quality of the coach-athlete relationship at time point 2 would mediate athletes’ use of communication strategies from time point 1 and time point 2 respectively. The findings highlight significant direct and indirect effects; specifically, the direct effects observed within the model indicate that communication at time 1 was positively and significantly related to coach-athlete relationship quality at time 2 as well as with communication at Time 2. Further, coach-athlete relationship quality at time 2 was positively and significantly related with communication at time 2. Together, these findings provide empirical support for the use of communication strategies in predicting the quality of the coach-athlete relationship over time as well as illustrating longitudinal associations between the use of communication strategies across the two time points. Specifically, when athletes perceive themselves to be using communication strategies aiming to help, facilitate, and comfort (i.e., support), as well as demonstrate their drive, effort, and energy for training (i.e., motivation), in addition to identifying, discussing, and resolving conflicts (i.e., conflict management), they are more likely to experience trusting, committed, and cooperative relationships over time. Together, these findings extend study 1 as well as previous research findings (Martindale et al., 2007; Rhind and Jowett, 2011, 2012) by investigating the temporal relationships between communication strategies and perceptions of relationship quality. Thus, the present study illustrates how relationship maintenance through communication (as defined by support, motivation, and conflict management strategies) leads to effective coach-athlete relationships over time. Additionally, the present findings support Rhind and Jowett’s (2012) proposal that the use of strategies enhance relationship quality and relationship quality in turn enhances the use of communication strategies. Indeed, the present study lends support to this contention in that communication strategies and relationship quality are reciprocally associated.

The main purpose of this study was to examine relationship quality at time point 2 as a mediator between athletes’ use of communication strategies at time point 1 and at time point 2. We found significant indirect effects for relationship quality at time point two on the associations between athletes use of communication strategies at time point 1 and time point 2, thus supporting mediation. Researchers (e.g., Jowett and Poczwardowski, 2007; Gilbert, 2017; Guerrero et al., 2018) have acknowledged that communication not only is central to coach-athlete relationships regardless of the type of sport, level of performance, or cultural backgrounds of the relationship members but it has also the capacity to function as both an antecedent and consequent variable of relationships. As early as 1976, when relationship research was at its infancy, Miller stated “Understanding the interpersonal communication process demands an understanding of the symbiotic relationship between communication and relational development: communication influences relational development and in turn (or simultaneously) relational development influences the nature of the communication between parties to the relationship” (p. 15).

Overall, these findings provide an original contribution to the literature and extend previous research by examining the role of communication strategies as defined by motivation, support and conflict management, and relationship quality as defined by the 3Cs over a period of time. Our findings highlight that athletes’ communication strategies are important and may act as the fuel that feeds the relationship by powering, energizing, and driving it forward (Jowett and Shanmugam, 2016). That said, the characteristics that describe the quality of the coach-athlete relationship (i.e., closeness, commitment, and complementarity) may also serve as strategies themselves to maintain and further enhance communication (Rhind and Jowett, 2012). A longitudinal empirical investigation that focuses on examining an intervention-based program informed by the properties that connect (3Cs) coaches and athletes and by the strategies of communication that coaches and athletes use may help us more readily unravel their unique effectiveness and predictive power relative to important outcomes (performance and satisfaction). In fact, their combined effects may be stronger predictors that either of them separately. This conjecture warrants investigation.

From an applied perspective, it is important that coaches and athletes remain sensitive to how one another relate and communicate. Raising awareness of the central role of relationships and communication should be a primary concern in training that aims to upskill coaches and athletes. Moreover, the development of interpersonal skills should also be central in such training programs. The objective of such training would be to enhance specific interpersonal skills that promote relational properties such as trust, respect, appreciation, commitment, sacrifice, accommodation, cooperation, responsiveness and the like as well as communication strategies including support, motivation and conflict management to mention a few. Relationship and communication-related training would provide a sound platform for the creation of social environments that are positive, safe and empowering within which both coaches and athletes experience success and satisfaction.

Whilst this study presents strengths, it is not without its limitations. First, with 107 athletes, the sample was relatively small and the study may have been deficient in statistical power to detect smaller effects (Cohen, 1992). Second, although longitudinal correlational studies can establish temporal relationships, they can only suggest and not establish causal relationships (Taris, 2000). In light of this, the findings of the present study may guide future relationship-based interventions that establish evidence based causal relationships. Third, communication was represented as a global construct in study 2 (unlike study 1), that consisted of the support, motivation, and conflict management dimensions of the COMPASS model (Rhind and Jowett, 2010); this approach may have failed to capture the influence of alternative communication strategies beyond those represented within the COMPASS model. Consequently, future studies may investigate longitudinal mediation effects with a variety of the communication dimensions within and beyond the COMPASS model as different strategies may vary in terms of their role and importance (Rhind and Jowett, 2012). Finally, it is important to note that like study 1, we collected data from predominantly club level, adult aged athletes; further research is merited to explore coach-athlete dyads while considering their individual difference characteristics (i.e., gender, age) relational (i.e., gender composition of dyad) and situational factors (i.e., level of competition). Dyadic data are of particular importance for future research given that coaches and athletes in two-person relationships do not interact and communicate in isolation.

## Summary Discussion

Although the role of coach-athlete relationships in sport has been widely recognized within the last two decades (Wylleman, 2000; Jowett, 2017), only limited research has examined the role of communication strategies within the context of the coach-athlete relationship (see, e.g., Rhind and Jowett, 2012). Previous research has found good quality relationships to be positively associated with an array of sporting outcomes (see Jowett and Shanmugam, 2016 for a full review) and negatively associated with interpersonal conflict (Jowett, 2009a; Wachsmuth et al., 2017). Nonetheless, there is dearth of research that examines the mechanisms by which the quality of the coach-athlete relationship associates with important outcomes. Communication has been conceptualized as a psychological process that mediates this association (Jowett and Poczwardowski, 2007). Indeed, our findings indicated that communication strategies (support, motivation, and conflict management) can help transfer the effects of quality coach-athlete relationships onto athletes’ satisfaction with their sporting experiences. Moreover, the findings from both studies and when paying attention to the direct effects show that good quality relationships characterized by trust, commitment and cooperation create a social environment within which athletes more readily engage in communication strategies. That said, it is important to highlight that communication within both study 1 and study 2 was represented by communication “strategies” employed to promote better quality relationships. Whilst this has been an important development in our research and for practice, communication also encompasses other components. In addition to the content of communication, future research should also pay attention to examining the emotional component (i.e., how/expression/affect) of communication within the coach-athlete relationship.

The second study advances theory by demonstrating temporal patterns across time. It would appear that both relationship quality and communication mutually influence one another and this reciprocal association may be vital processes for the achievement of important outcomes related to performance (training and competence) and well-being (satisfaction). Future longitudinal intervention-based research may build on the quantitative data presented in this paper by obtaining qualitative data through diaries and observational techniques (e.g., Allan et al., 2016) allowing for more objective assessment of the generated usage of such strategies, in addition to examining their effectiveness. In summary, communication is a significant factor not only for the development of high quality coach-athlete relationships but also for continuous sport participation that is both rewarding and satisfying. Our findings provide novel contributions for both theory and practice. Future research should consider these findings as building blocks for intervention programs that utilize relationship and communication as psychological process for behavioral change.

## Data Availability Statement

The datasets generated for this study are available on request to the corresponding author.

## Ethics Statement

The studies involving human participants were reviewed and approved by Loughborough University. The patients/participants provided their written informed consent to participate in this study.

## Author Contributions

LD designed study 1 and study 2, collected data and prepared and wrote the manuscript. SJ designed both study 1 and study 2 together with LD and collected data. SJ inputted into the manuscript. ST analyzed the data and inputted into the results section of the manuscript.

## Conflict of Interest

The authors declare that the research was conducted in the absence of any commercial or financial relationships that could be construed as a potential conflict of interest.
